# Transcriptome sequencing of transgenic poplar (*Populus *× *euramericana *'Guariento') expressing multiple resistance genes

**DOI:** 10.1186/1471-2156-15-S1-S7

**Published:** 2014-06-20

**Authors:** Weixi Zhang, Yanguang Chu, Changjun Ding, Bingyu Zhang, Qinjun Huang, Zanmin Hu, Rongfeng Huang, Yingchuan Tian, Xiaohua Su

**Affiliations:** 1 State Key Laboratory of Tree Genetics and Breeding, Research Institute of Forestry, Chinese Academy of Forestry, Beijing 100091, China; 2 Key Laboratory of Tree Breeding and Cultivation of State Forestry Administration, Beijing 100091, China; 3 Institute of Genetics and Developmental Biology, Chinese Academy of Sciences, Beijing 100101, China; 4 Biotechnology Research Institute, Chinese Academy of Agricultural Sciences, Beijing 100081, China; 5 Institute of Microbiology, Chinese Academy of Sciences, Beijing 100101, China

**Keywords:** transgenic poplar, stress resistance, transcriptome, functional annotation, *Populus × euramericana* 'Guariento', RNA-Seq

## Abstract

**Background:**

Transgenic poplar (*Populus × euramericana '*Guariento*'*) plants harboring five exogenous, stress-related genes exhibit increased tolerance to multiple stresses including drought, salt, waterlogging, and insect feeding, but the complex mechanisms underlying stress tolerance in these plants have not been elucidated. Here, we analyzed the differences in the transcriptomes of the transgenic poplar line D5-20 and the non-transgenic line D5-0 using high-throughput transcriptome sequencing techniques and elucidated the functions of the differentially expressed genes using various functional annotation methods.

**Results:**

We generated 11.80 Gb of sequencing data containing 63, 430, 901 sequences, with an average length of 200 bp. The processed sequences were mapped to reference genome sequences of *Populus trichocarpa*. An average of 62.30% and 61.48% sequences could be aligned with the reference genomes for D5-20 and D5-0, respectively. We detected 11,352 (D5-20) and 11,372 expressed genes (D5-0), 7,624 (56.61%; D5-20) and 7,453 (65.54%; D5-0) of which could be functionally annotated. A total of 782 differentially expressed genes in D5-20 were identified compared with D5-0, including 628 up-regulated and 154 down-regulated genes. In addition, 196 genes with putative functions related to stress responses were also annotated. Gene Ontology (GO) analysis revealed that 346 differentially expressed genes are mainly involved in 67 biological functions, such as DNA binding and nucleus. KEGG annotation revealed that 36 genes (21 up-regulated and 15 down-regulated) were enriched in 51 biological pathways, 9 of which are linked to glucose metabolism. KOG functional classification revealed that 475 genes were enriched in 23 types of KOG functions.

**Conclusion:**

These results suggest that the transferred exogenous genes altered the expression of stress (biotic and abiotic) response genes, which were distributed in different metabolic pathways and were linked to some extent. Our results provide a theoretic basis for investigating the functional mechanisms of exogenous genes in transgenic plants.

## Background

Forest trees are renewable natural resources that are vital to the balance of the terrestrial ecosystem and have important commercial applications, including timber wood, paper and pulp, and biofuel production. The growth and development of forest trees are frequently challenged by biotic (such as pests and diseases) and abiotic (such as drought, soil salinity, and flooding) stresses, although natural forests have evolved a certain ability to cope with these adverse environmental factors. Genetically engineered plants have been developed in a wide range of tree species, and numerous transgenic clones with improved traits have been generated, some of which have undergone field trials to the environmental release stage; the genetic stability of genetically modified forests has been verified in field trials [1]. Forest molecular breeding research has been greatly enhanced by the completion of genome-wide sequencing projects, the development of genome-wide chips, and the establishment of genetic maps in poplar.

Genes of interest that can be used in the genetic engineering of abiotic stress-resistant plants can be divided into two categories: 1) genes that directly respond to stress, which mainly include genes encoding functional proteins that protect the cell against stress damage, such as enzymes for the synthesis of osmolytes, and enzymes for the removal of active oxygen; 2) genes that regulate genetic expression and signal transduction under stress, which mainly include genes encoding transcription factors, protein kinases, and others. For example, some genes are involved in the expression of osmolytes, such as proline, glycine, betaine, sucrose, fructan, and so on, and they help plants accumulate osmolytes to maintain the osmotic balance and body moisture levels, which improves the drought tolerance of plants under drought and salt stress [2]. Indeed, the exogenous expression of the fructan synthase gene (*sacB*) and trehalose-6-phosphate synthase genes (*otsB, otsA*, and *TPS1*) can improve the drought-resistance, salt-tolerance, and low-temperature-resistance of transgenic poplar [3], rice [4], spinach beet [5], and tobacco [6,7]. Transporter genes can help alter the ionic and osmotic balance in plants by up-regulating the expression levels of proteins to control ion transport under abiotic stress [8]; for example, *HAL1 *expression improves the salt tolerance of transgenic tomatoes [9]. Vitreoscilla hemoglobin (VHb), which has obligate aerobic properties, can be synthesized at substantial levels in plants under hypoxic conditions [10]; VHb overexpression can improve cellular oxygen ion levels and terminal oxidase activity under waterlogging stress. VHb gene expression can facilitate the growth of transgenic *Nicotiana tabacum *[11,12], *Datura innoxia *[13], and *Petunia hybrida *Vilm [14].

Transcriptional regulation is a major stress response mechanism in plants. Under stress conditions, transcription factors involved in stress resistance can regulate the simultaneous expression of multiple stress-resistance genes and genes involved in the transport of stress signals [8]. Therefore, regulating the expression of transcription factors has been proposed as a way to improve the stress resistance of plants. Numerous studies have shown that members of the MYB, MYC, ERF, bZIP, and WRKY transcription factor families are involved in stress response regulation [15]. For example, overexpression of CaPF1 [16], AtDREB1A [17,18], CBF3/DREB1A [18], SNAC1 [19], and others can increase the stress resistance of transgenic plants. Protein kinase plays an important role in responses to environmental changes and signal transduction in plants. For example, the expression of calcium-dependent and calmodulin-independent protein kinase (CDPK) genes, including *NtCDPK4 *[20], *OsCPK6, OsCPK13, OsCPK17*, and *OsCPK25 *[21], increases under drought, high salt, or low temperature stress, and tolerance to drought and high salt stress is significantly improved in transgenic *Arabidopsis thaliana *harboring *AtCPK23 *[22]. Receptor-like kinases (RLKs) on cell membranes can sense external stimuli and are involved in intracellular signal transduction. Overexpression of RLK genes, e.g., *CaMRP1 *[23] and *StLRPK1 *[24], can significantly improve the survival ability of plants under environmental stress conditions. Additionally, studies involving biotic stress resistance genes have shown that *Bacillus thuringiensis *(Bt) expresses proteins that are toxic to a variety of insects, and these proteins have been broadly applied in anti-insect plant research [25] . Protease inhibitors, such as cysteine proteinase inhibitor (OCI) [26] and cowpea trypsin inhibitor (CPTI) [27], have been used independently or in conjunction with *Bt *genes to significantly increase the insect resistance of important agricultural plants [28]. In addition, overexpression of the chitinase gene can significantly increase disease resistance in plants [29].

Numerous transgenic plants that are resistant to abiotic stress (drought, waterlogging, salinity) and biotic stress (disease, pests) have been produced using genetic transformation techniques, and extensive research on their exogenous gene expression has been performed [2,8,30,31]. Nevertheless, many agronomic traits, such as insect and salt resistance, are usually jointly controlled by different genes. Therefore, some studies have focused on plant agronomic trait improvement and molecular breeding by means of gene connection or co-transformation to produce new, excellent varieties. However, most genetic transformation is limited to 1-3 major gene(s) in the same or related pathways. For example, Ye *et al*. [32] co-transformed rice with two major plant enzyme genes involved in regulating the synthesis of previtamin A or lycopene in endosperm chromatophore, producing transgenic plants that synthesize B-carotene in the endosperm, namely, golden rice. Most studies examining how transgenic plants respond to stress have been limited to examining to the expression of exogenous genes and the accumulation of metabolic substances that are directly related to stress responses. Few studies have examined the expression of stress resistance genes in plants on a genome-wide level. For example, Chan *et al*. [33] examined the transcriptome of *Arabidopsis thaliana *harboring the mannose-6-phosphate reductase gene *M6PR *an found that this gene activates the downstream abscisic acid (ABA) pathway by up-regulating the expression of ABA receptor genes (*PYL4, PYL5*, and *PYL6*) and down-regulating the expression of protein phosphatase 2C genes (*ABI1 *and *ABI2*) under salt stress. Maria *et al*.[34] found that half of the differentially expressed genes in the transcriptome of transgenic rice (compared to non-transgenic rice) are linked to transferred, exogenous genes. Coll *et al*.[35] evaluated the transcriptome differences between the commercial transgenic maize line MON810 and non-transgenic maize (in a 1/3 maize gene expression level) and found few differentially expressed genes between these lines. However, the different expression levels between transcriptomes of transgenic and non-transgenic plants have not been discovered.

*Populus *× *euramericana *is an interspecific hybrid produced from the cross of *Populus nigra *and *Populus deltoides*. Many *P*. × *euramericana *clones have been commercialized and used in forestry production and to promote ecosystem stability. We previously generated transgenic *Populus *× *euramericana '*Guariento*' *harboring five effect genes [36]. These five genes include the following: *Vgb*, encoding aerobic Vitreoscilla hemoglobin (VHb); *SacB*, encoding levansucrase, which is involved in *Bacillus subtilis *fructan biosynthesis; *BtCry3A*, encoding the *Bt *endotoxin from Coleoptera; *OC-I*, an anti-insect gene, encoding rice cystatin; and *JERF36*, a tomato gene, encoding the jasmonate (JA)/ethylene (ET) response factor protein. Greenhouse/laboratory experiments and field trials of two transgenic clones (D5-20 and D5-21), including stress (drought, soil salinity, and flooding) resistance and field experiments, have shown that the transgenic plants have increased tolerance to multiple stresses including drought, salt, waterlogging and insect feeding [37]. In the present study, we intensively sequenced the transgenic clone D5-20 and the non-transgenic clone D5-0 using high-throughput transcriptome sequencing techniques. We then examined the expression of differentially expressed genes in transgenic vs. non-transgenic lines at the genome-wide level, and we explored the mechanisms used by the exogenous genes in the transgenic plants.

## Results

### Illumina sequencing and alignment to the reference genome

Two cDNA libraries, derived from D5-20 (transgenic) and D5-0 (non-transgenic) lines, were sequenced using Illumina HiSeq 2000 high-throughput sequencing. A total of 63, 430, 901 sequences were generated, with an average length of 200 bp, yielding approximately 11.80 Gb of sequencing data (Table 1). More than 30, 000, 000 sequencing reads were generated from each sample. After removing the primer and adaptor sequences and performing quality inspection of the 3'-termini in the sequenced fragments, the sequenced fragments with reliable quality were selected, the basic groups of lower quality were removed from the 3' termini, and the sequences with high sequencing quality (comprising 99% of the raw data) were subjected to further analysis. The processed sequencing reads were mapping to reference genome sequences of *Populus trichocarpa*. Of the total reads, 61.89% could be aligned with the reference genomes; the remaining 31.77% were unaligned (Table 1). This result is mainly due to the dissimilar genera of Populus and the significant genetic differences and high degree of differentiation between dissimilar species.

**Table 1:** Comparative statistics of sequencing data output and reference genomes

	Line	
	
	D5-20	D5-0
Total reads	32359140	30079761
Filtered reads	42199	39521
Remained reads	32342328	30064336
Aligned reads	20147954 (62.30%)	18482966 (61.48%)
Unaligned reads	11853904 (36.65%)	11290950 (37.56%)
Misaligned reads	340470 (1.05%)	290420 (0.97%)

### Global analysis of gene expression

To obtain robust information about the biological differences among samples, it is important to utilize biological replications. Here, we utilized a reproducible experimental design (Group A: Aa + A2 and so on; two groups in total) to analyze the expression profiles (a total of three alignment groups); variances were calculated at the levels of genes and gene isoforms separately to obtain typical samples for intensive sequencing analysis. The results presented below highlight the differences in gene expression levels between D5-20 and D5-0.

We calculated the Fragments Per Kilobase of transcript per Million fragments mapped (FPKM) values to indicate the gene expression levels within reference gene regions in standard read mode using Cufflink software. The FPKM approach is consistent with the RPKM calculation method, and it can be used to eliminate the influence of gene length and sequencing amount on the calculation of gene expression levels. Calculated gene expression levels can be directly applied when comparing the gene expression differences between species [38]. The expression of mRNA has two notable features: heterogeneity and redundancy. A small portion of mRNA is highly expressed, while most mRNA is expressed at lower levels. Therefore, data concerning the gene expression density distribution within samples can be used to evaluate the normality of RNA-Seq data. Additional file 1 shows that the sequencing data in the present study are distributed normally and can be used for further analysis.

**Figure 1:**
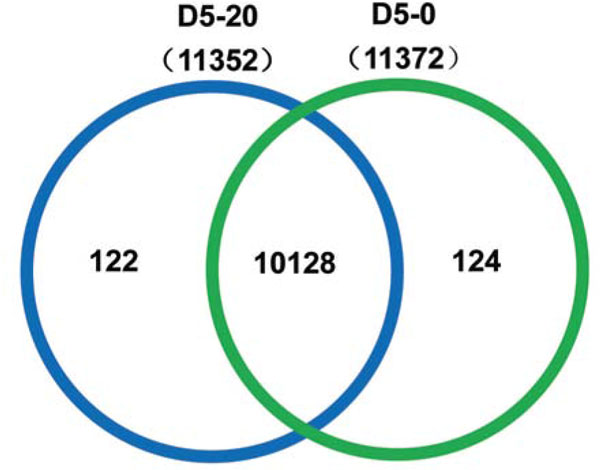
**Venn diagram showing the expression of D5-20 and D5-0 genes**.

A total of 11,352 genes (D5-20) and 11,372 genes (D5-0) were detected within the ranging from 150 bp to ≥ 2,000 bp. Analysis of this dataset (Table 2) showed that 4,036 expressed genes in the 500 to 1,000 bp range accounted for 32.04% (3,292) of expressed genes, while 26.14% of expressed genes ranged from 1,000 to 1,500 bp, and the number of expressed genes >2,000 bp was minimal, accounting for only 12.46% of expressed genes. A total of 12,596 genes were predicted after the removal of partial overlapping sequences. The relationship of expressed genes between the two samples is illustrated in Figure 1, wChich shows the distribution of expressed genes from D5-20 and D5-0. Among these genes, 10,128 genes were co-expressed in D5-20 and D5-0, 1,244 genes were expressed only in D5-0, and 1,224 genes were specifically expressed in D5-20.

**Table 2:** Distribution of gene sequences detected in *P*. × *euramericana 'Guariento' via RNA-Seq*

Gene length (bp)	Number of sequences	Percentage (%)
150-500	2080	16.51
500-1000	4036	32.04
1000-1500	3292	26.14
1500-2000	1619	12.85
>2000	1569	12.46
Total	12596	100

We examined the gene sequences using the KOG annotation system (http://genome.jgi.doe.gov/ Tutorial/tutorial/kog.html). Gene functional annotation information was provided for expressed *P. × euramericana '*Guariento' genes. We found that 7,624 genes (D5-20) and 7,453 genes (D5-0) were annotated in the two specimens, respectively, accounting for 56.61% (11,352) and 65.54 % (11,372) of the total number of genes.

### Analysis of differentially expressed genes in transgenic clone compared with nontransgenic poplar

**Figure 2:**
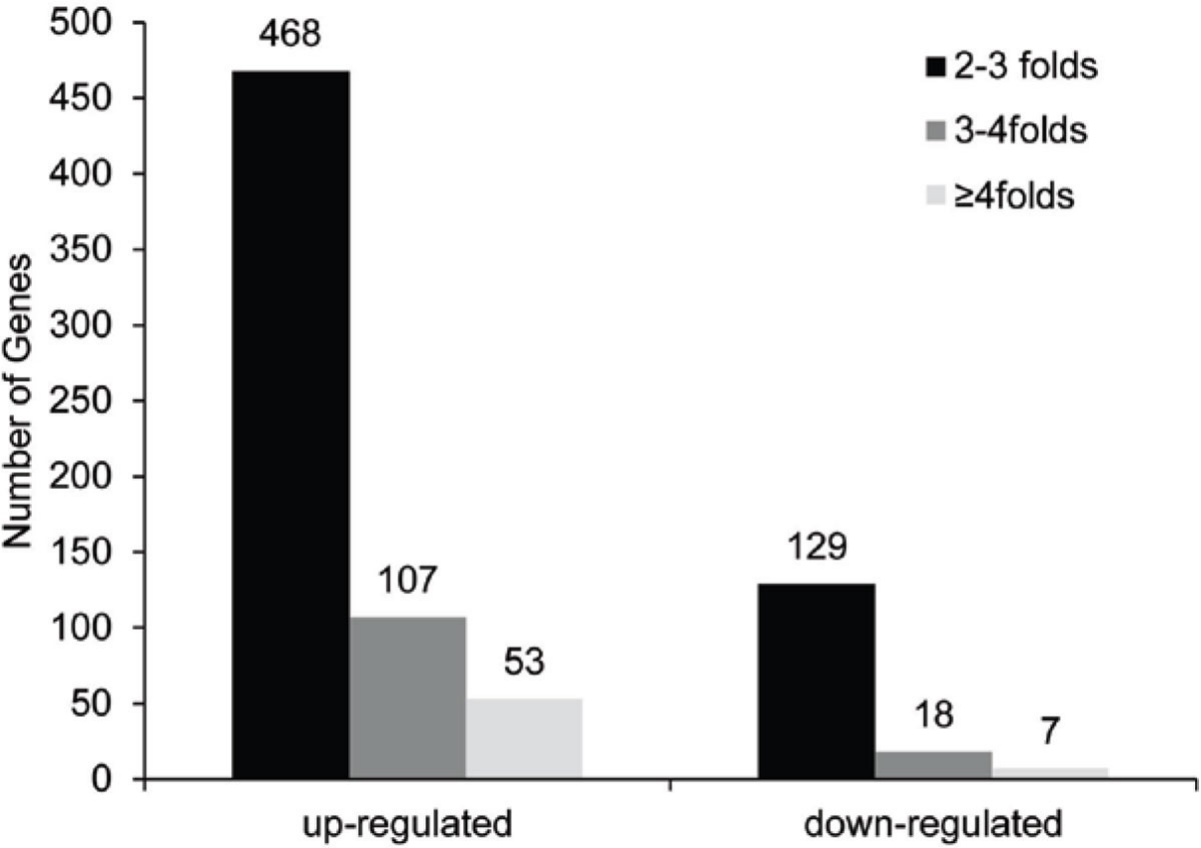
**Differences in gene expression between transgenic and nontransgenic *Populus × euramericana *'Guariento'**.

To identify differentially expressed genes between the transgenic (D5-20) and non-transgenic (D5-0) clones, the gene expression profiles in the two samples were compared and analyzed. The deviation of fragments in replicate samples was modeled using the negative Bernoulli distribution method. Fragments were evaluated according to threshold *q *value ≤ 0.05 after multiple hypothesis testing. Fold-change values between samples were calculated according to FPKM values. Values of "| log2 Ratio | ≥ 1 and false discovery rate (FDR) ≤ 0.05" were used as the threshold to assess the significance of differential gene expression. A total of 782 genes with significantly altered expression were detected between D5-20 and D5-0. The majority of these genes (628, 80.31%) showed up-regulated expression in D5-20, among which 468, 107, and 53 genes displayed 2-3-, 3-4-, and more than 4-fold higher expression in D5-20 than in D5-0, respectively. The remaining 154 genes (19.69%) were down-regulated, including 129, 18, and seven genes showing 2-3-, 3-4-, and more than 4-fold lower expression in D5-20 than in D5-0, respectively (Figure 2). These results suggest that the overexpression of exogenous genes results in remarkable changes in the transcriptome of transgenic poplar, which primarily includes the increased expression of hundreds of genes.

### Functional annotation of differentially expressed genes

**Figure 3:**
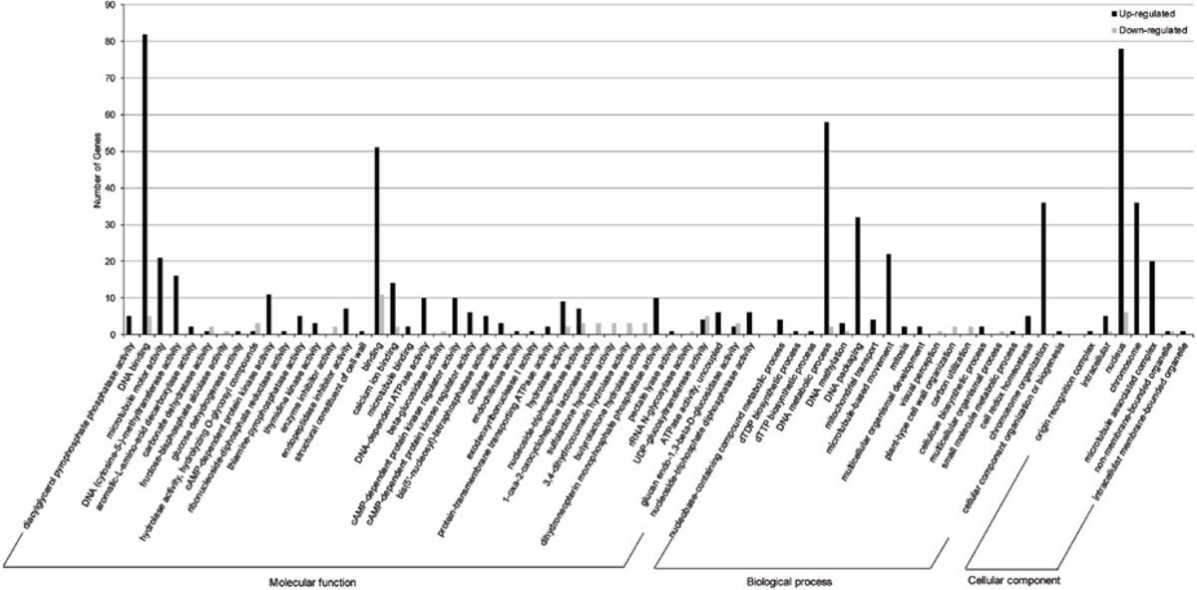
**GO classification map of differentially expressed genes**.

To better understand the functions of differentially expressed genes between transgenic and nontransgenic poplar, we performed Gene Ontology (GO) category enrichment analysis using Fisher's test, with *p *value ≤ 0.01 as a threshold. The results show that 346 differentially expressed genes (44.25%) could be categorized into 69 functional groups. The three main categories (molecular function, biological process, and cellular component) of the GO classification contained 44, 18, and seven functional groups, respectively (Figure 3 and Additional file 2). Among these groups, most of the differentially expressed genes were classified into the terms DNA binding (GO: 0003677), DNA metabolic process (GO: 0006259), and nucleus (GO: 0005634) in each of the three main categories, respectively. Additionally, a high percentage of differentially expressed genes mapped to functional groups of binding (GO: 0005488), chromosome organization (GO: 0051276), chromosome (GO: 0005694), DNA packaging (GO: 0006323), microtubule-based movement (GO: 0007018), microtubule motor activity (GO: 0003777), and microtubule associated complex (GO: 0005875) processes.

**Figure 4:**
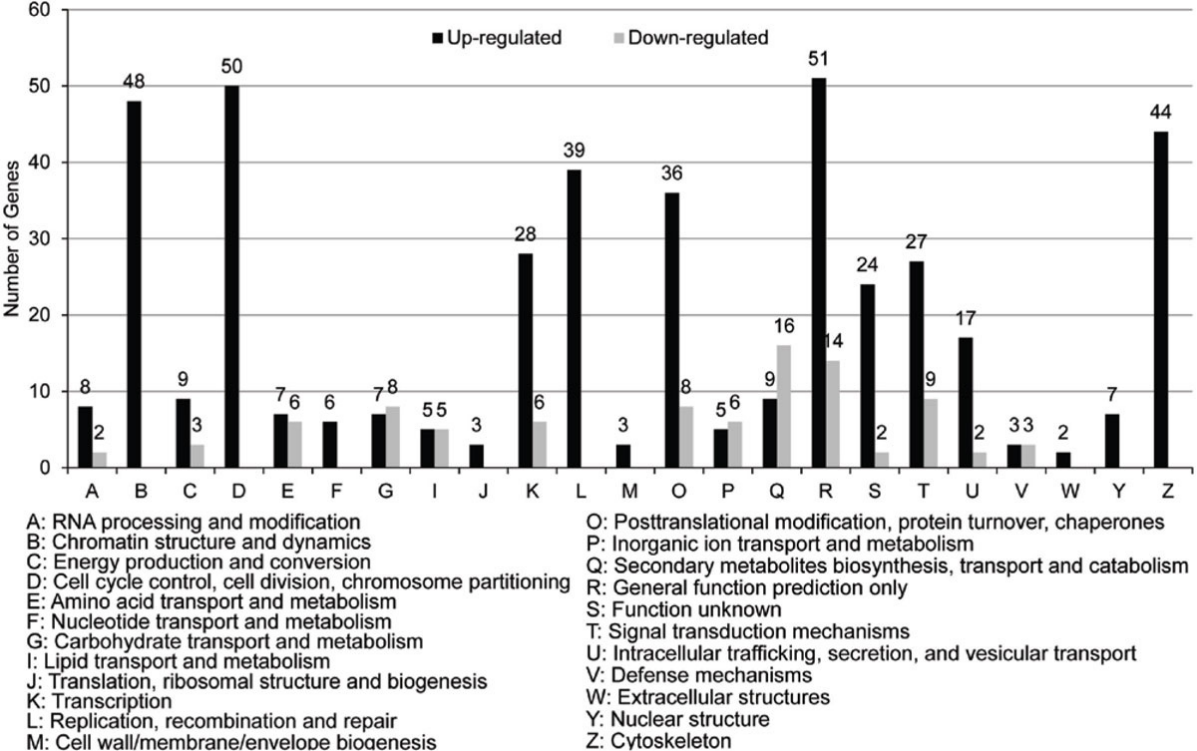
**KOG classification and analysis of differentially expressed genes**.

To examine the functional distribution characteristics of the differentially expressed genes more closely, KOG classifications of differentially expressed genes were performed according to the whole-genome annotation of *P. trichocarpa *(http://www.phytozome.net/poplar). KOGs are clusters of orthologous groups from complete eukaryotic genomes. A total of 475 out of 782 differentially expressed genes were enriched in 23 KOG categories (Figure 4; Additional file 3). The R category (general function prediction only) was the most highly enriched category, with 65 genes, accounting for 13.68% of the total number of genes. The D category (Cell cycle control, cell division, chromosome partitioning) contained the second largest number of genes (50), accounting for 10.53% of the total number of genes. There were 48 differentially expressed genes in the B (Chromatin structure and dynamics) categories, accounting for 10.11% of the genes. Additionally, 34-44 differentially expressed genes in the, K (Transcription), L (replication, recombination, and repair), O (Posttranslational modification, protein turnover, chaperones), T (Signal transduction mechanisms), and Z (Cytoskeleton) categories accounted for 5.05-9.26% of the total number of genes. All of the differentially expressed genes in the B and F (Nucleotide transport and metabolism), M (Cell wall/membrane/envelope biogenesis), W (Extracellular structures), Y (Nuclear structure), and Z categories were up-regulated; A large proportion of differentially expressed genes in the A (RNA processing and modification), C, K, O, R, S (Function unknown), T, and U(Intracellular trafficking, secretion, and vesicular transport) categories were up-regulated. A large proportion of differentially expressed genes in the Q (Secondary metabolites biosynthesis, transport and catabolism) categories were down-regulated.

### Biological pathway analysis of differentially expressed genes

We performed pathway enrichment analysis to further investigate the biological functions of differentially expressed genes. We mapped all of these genes to reference pathways in the Kyoto Encyclopedia of Genes and Genomes (KEGG) database (http://www.genome.ad.jp/kegg/) to identify the biological pathways in which the genes may be involved. Of the 782 differentially expressed genes, 36 genes (21 up-regulated and 15 down-regulated) could be assigned to 51 KEGG pathways (Additional file 4). Notably, 18 genes were assigned to 9 glucose metabolic pathways. Among these genes, 13 (seven up- and six down-regulated), four (two up- and two down-regulated), and two (down-regulated) were predicted to be involved in "starch and sucrose metabolic (KO00500)", "Pentose and glucuronate interconversions (KO00040)", and "glycolysis/gluconeogenesis (KO00010)". Six other down-regulated genes encoding fructose-bisphosphate aldolases were sorted into glucose metabolic-related pathways (KO00010, KO00030, and KO00051). Additionally, 10 genes were linked to seven KEGG pathways related to amino acid metabolism, including two up-regulated genes involved in "cysteine and methionine metabolism" (KO00270). Moreover, we also identified one and two up-regulated genes that could be associated with "glutathione metabolic (KO00480)" and "Drug metabolism-other enzymes (KO00983)", respectively (Additional file 5). These KEGG annotations provide important clues for investigating specific biological processes that can be influenced by the expression of foreign genes in poplar transformed with multiple genes.

### Stress response-related genes

We performed homologous protein alignment and annotation analysis for differentially expressed genes using SwissProt and the Uniport nonredundant protein database. The translated protein sequences encoded by the differentially expressed genes were aligned using Blastp analysis of protein databank sequences; the optimally aligned protein with *e *value < 1E-5 were used to identify the candidate names of differentially expressed genes/proteins. A total of 730 significantly differentially expressed genes could be annotated to homologous proteins, and the candidate names of 492 differentially expressed genes/proteins were identified. Among the 492 genes, 196 were considered to be putative stress response genes based on GO and KOG analysis. These stress-related genes could be sorted into seven major classes (Additional file 6); of these, serine/threonine protein kinase (39 genes), signaling molecule (27), and transcription factor (34) were the three classes with largest number of genes. Other classes including genes involved in oxygen metabolism, transporters, molecular chaperones, AAA+-type ATPases, and chitinases were also identified. Interestingly, members of several gene families were overrepresented among the stress-related genes, such as 17 up-regulated genes encoding receptor-like kinases, 11 encoding ubiquitin-protein ligases (nine up- and two down-regulated), 14 encoding AP2/ERF transcription factors (11 up- and three down-regulated), 11 encoding cytochrome P450 enzymes (four up- and seven down-regulated), and nine up-regulated genes encoding esterase/lipases.

### Phylogenetic analysis of stress-related gene

**Figure 5:**
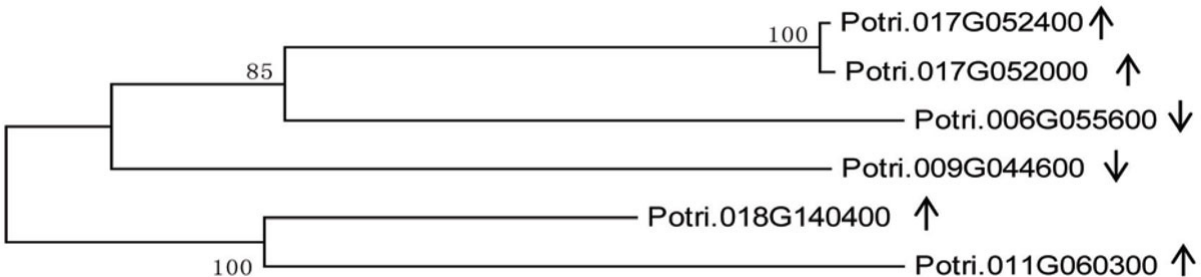
**Phylogenetic analysis of UDP-glucuronosyl/UDP glycosyltransferase genes**. ↑, up-regulated genes; ↓, down-regulated gene.

Phylogenetic analysis revealed that seven differentially expressed genes are transcription factor genes in the ERF family in poplar [39] (including six belonging to Group B1 and one belonging to Group B3), while three differentially expressed genes are transcription factor genes in the DREB family, and one differentially expressed gene encodes a transcription factor in the AP2 family (Group A2) among up-regulated genes. Among down-regulated genes, one differentially expressed gene is a transcription factor gene in the DREB family (Group A4) and two differentially expressed genes are transcription factor genes in AP2 family. Phylogenetic analysis of six UDP-glucuronosyl/UDP glycosyltransferase genes revealed that two up-regulated genes (Potri.017G052400, Potri.017G052000) have the same amino acid sequence and are in one group, while two other up-regulated genes (Potri.018G140400, Potri.011G060300) and two down-regulated genes (Potri.009G044600, Potri.006G055600) are in the other groups (Figure 5).

### Validation of RNA-Seq results by qRT-RCR

**Figure 6:**
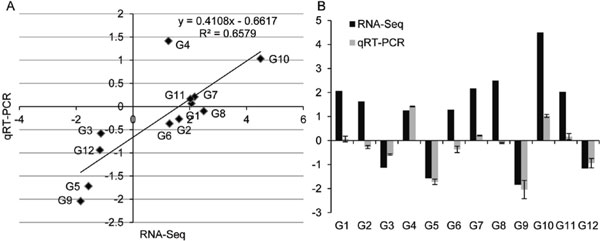
**qRT-PCR validation of differentially expressed genes of transgenic poplar**. A, correlation of fold change analyzed by RNA-Seq (x-axis) with data obtained using qRT-PCR (y-axis). B, Expression analysis of differentially expressed genes between RNA-Seq and qRT-PCR. G1-12 : Potri.005G223100, Potri.007G138100, Potri.003G139300, Potri.017G052000, Potri.006G055600, Potri.016G128300, Potri.006G105300, Potri.001G202100, Potri.009G129900, Potri.001G325800, Potri.006G112500, Potri.T056000.

To confirm the accuracy and reproducibility of the Illumina RNA-Seq results, quantitative real-time (qRT)-PCR was performed on 12 randomly selected genes with increased transcript abundance or decreased transcript abundance in transgenic poplar. The correlation between RNA-Seq and qRT-PCR data was evaluated using fold-change measurements. To compare fold changes, a scatter plot was generated using the log 2 fold change determined between RNA-Seq and qRT-PCR data, which is defined as ΔΔCT (for comparative threshold cycle). As shown in Figure 6A, the qRT-PCR results revealed that the expression trends of these genes showed significant similarity (R^2 ^= 0.64) with the RNA-Seq data (Figure 6A). This result reflects the accuracy and reproducibility of the RNA-Seq results. Of the 12 genes examined, the qRT-PCR expression data from nine genes showed similar trends to the RNA-Seq data (Figure 6B); among these, the fold changes of expression for four genes (Potri.017G052000, Potri.006G055600, Potri.009G129900, and Potri.T056000) were in complete agreement with the RNA-Seq data, while the fold changes of expression for five genes (Potri.005G223100, Potri.003G139300, Potri.006G105300, Potri.001G325800, and Potri.006G112500) were lower than those obtain by RNA-Seq. The remaining three genes (Potri.007G138100, Potri.016G128300, and Potri.001G202100) showed slightly lower levels of expression compared with the RNA-Seq data (Figure 6B).

**Figure 7:**
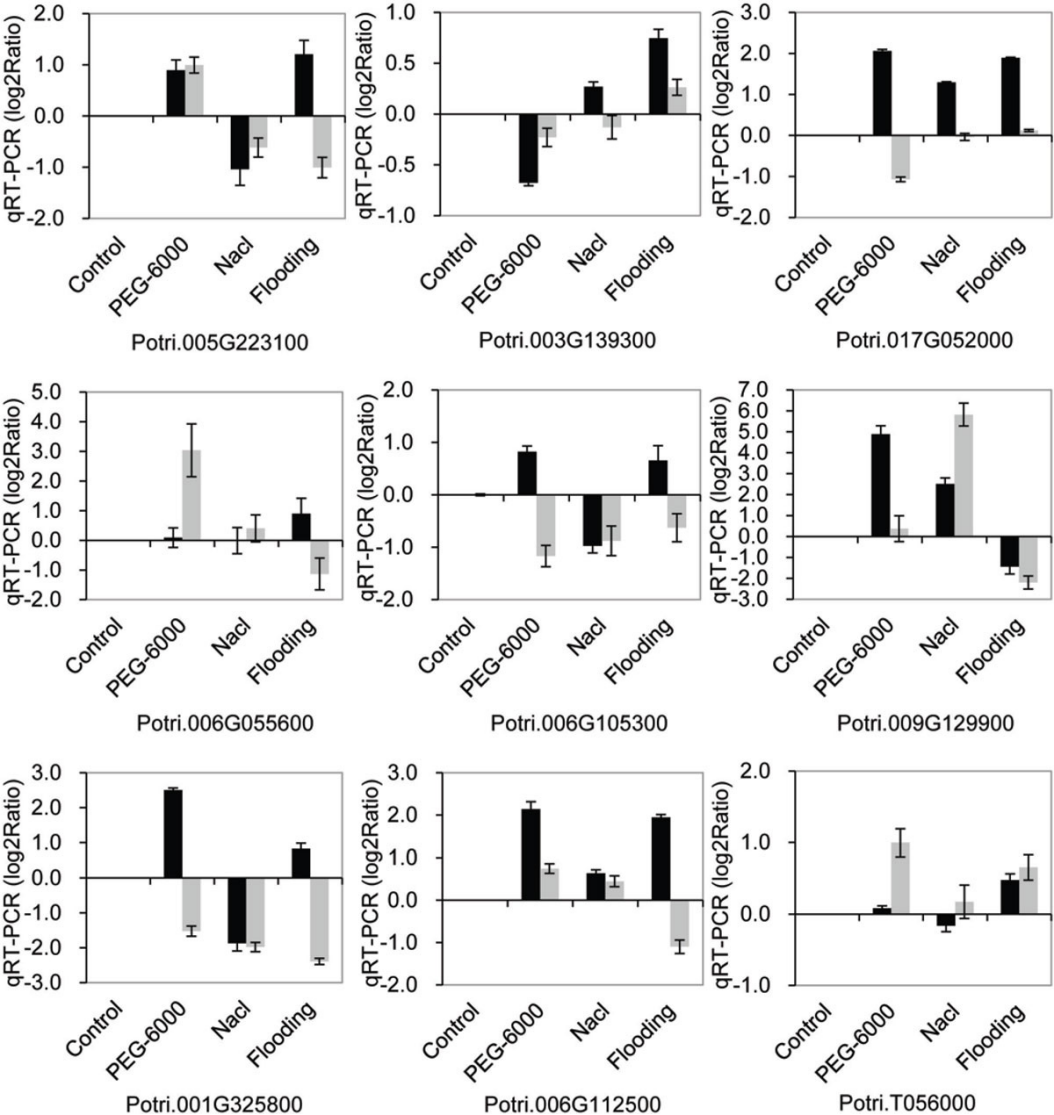
**Genes of transgenic and nontransgenic poplar expressed under drought, salt, and F stress examined by qRT-PCR**. The relative expression level was log2 Ratio, > 0 means up-regulated, = 0 means unregulated and < 0 means down-regulated. The black bar shows D5-0 vs. D5-0; D5-0 under non-stress conditions was used as a control for drought (PEG-6000), salt (Nacl), and Flooding stress. The gray bar shows D5-20 vs. D5-20; D5-20 under non-stress conditions was used as a control for drought, salt, and watering stress.

**Figure 8:**
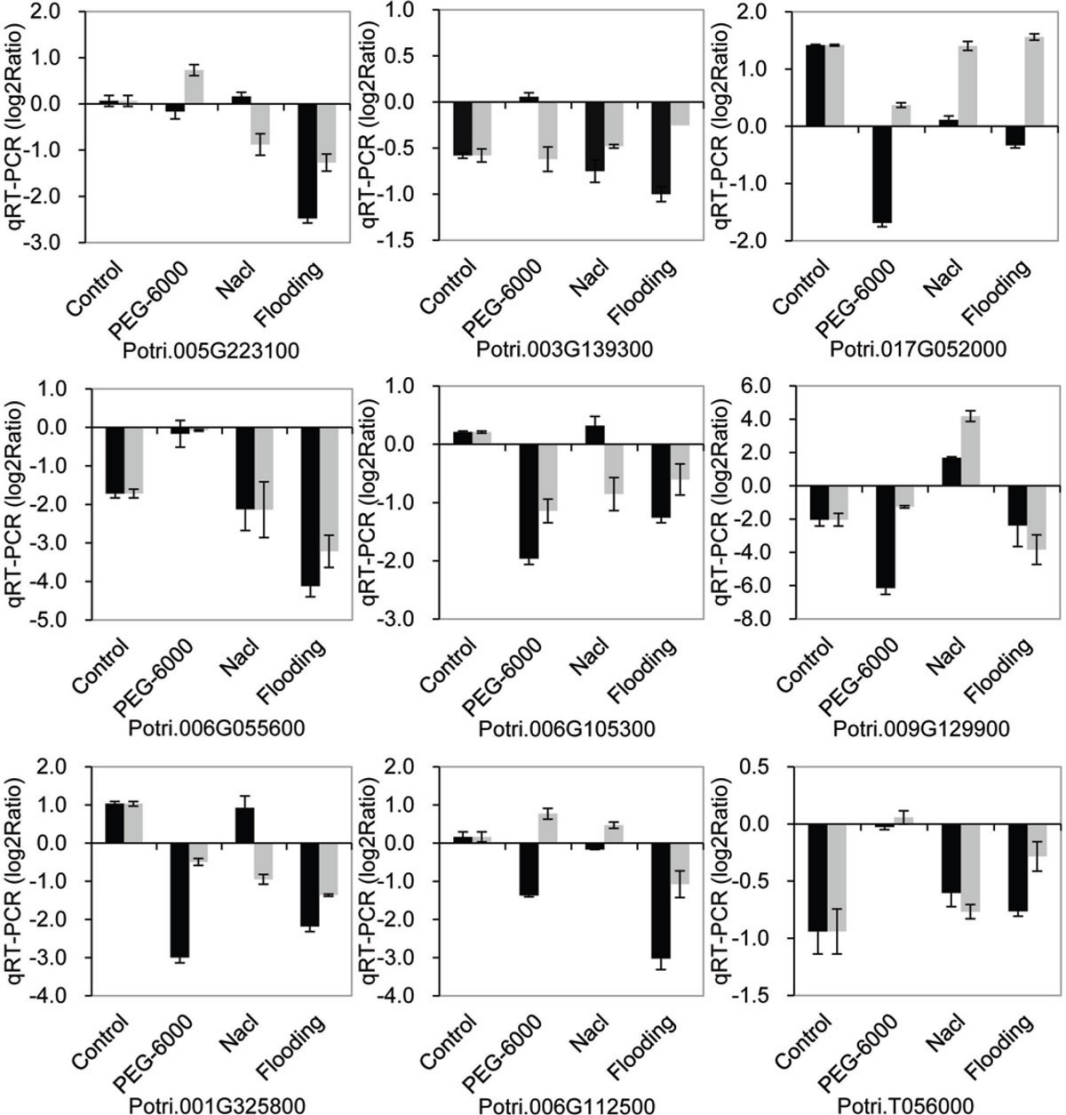
**qRT-PCR validation of differentially expressed genes of transgenic and nontransgenic poplar under drought, salt, and watering stress**. The relative expression level was log2 Ratio, > 0 means up-regulated, = 0 means unregulated and < 0 means down-regulated. The black bar shows D5-20 vs. D5-0; D5-0 was used as a control under drought (PEG-6000), salt (Nacl), and Flooding stress, respectively; the gray bar shows D5-20 vs. CD5-0; D5-0 under non-stress conditions was used as a control for drought, salt, and watering stress.

The expression of the nine genes that were in agreement with RNA-Seq data was investigated under drought, salt, and flooding stress by qRT-PCR. Under the same stress (drought, salt, or flooding) conditions (Figure 7), the trends of gene expression differed between transgenic and nontransgenic poplars; Potri.003G139300 and Potri.006G055600 showed similar expression trends under drought and watering stress, while Potri.005G223100, Potri.009G129900, and Potri.006G112500 showed similar expression trends under drought and salt stress. On the other hand, the expression patterns of these genes were not completely consistent between different stress conditions, while under drought, salt, or watering stress (Figure 8), three genes (Potri.003G139300, Potri.006G055600, and Potri.T056000) were down-regulated under all three stress conditions and exhibited a similar trend under non-stressed condition, suggesting that these three genes may be under negative regulation. The expression patterns of the remaining six genes under at least one stress treatment (drought, salt, or flooding), were similar to those observed in plants under non-stressed conditions.

## Discussion

We performed gene expression analysis of transgenic poplar using Illumina HiSeq 2000 paired-end sequencing (RNA-Seq). We have generated 63, 430, 901 sequence reads (200 bp in length) corresponding to 11.80 Gb of sequencing data. Approximately 73.33% of the sequences could be mapped onto the reference genome sequence of *P. trichocarp*a. In grape, a total of 59,372,544 sequences reads corresponding to 2.2 Gb of sequencing data were generated by RNA-seq sequencing from three developmental stages, and approximately 82.4% of the sequences were aligned with the reference genome (Pinot Noir 40024) [40]. This result suggests that significant genetic divergence exists in the genus *Populus*, and the degree of genomic difference in poplars may be higher than that in grape. Global analysis of gene expression revealed transcriptome reprogramming in transgenic poplar (D5-20) compared with the non-transgenic clone D5-0. A total of 782 genes were found to be differentially expressed, including 628 up-regulated (80.3%) and 154 down-regulated genes (19.7%), indicating that transcriptional activators play a major role of in the insertion/expression of the five transgenes in D5-20. Moreover, 197 putative biotic or abiotic stress-responsive genes were also identified. The data from RNA-Seq were validated by qRT-PCR analysis of 12 genes. The expression of nine out of 12 genes showed diverse trends under stress (drought, salt, or flooding) treatment, as revealed by qRT-PCR analysis (Figure 8), which may be associated with the introduction of exogenous genes and may involve different regulatory networks of these genes. Compared with non-transgenic poplar, the genes exhibited similar trends in expression under non-stressed conditions and under at least one stress condition, as revealed by qRT-PCR and RNA-Seq analysis. These genes may be involved in different regulatory networks under different stress conditions. These complex results suggested that there are complex interactions among multiple genes and the exogenously expressed genes. The magnitude of the number differentially expressed genes found in this study is similar to previously reported ranges for transgenic plants harboring single transformation constructs [34,41,42].

Fructans are involved in osmoregulation. Fructans accumulate under stress conditions as a result of the fructosyltransferase effect. The *SacB *in *Bacillus subtilis *encodes secreted levansucrase, which catalyzes the synthesis of fructans from substrate sucrose. The expression of *sacB *can increase plant cold and drought tolerance by significantly enhancing the accumulation of fructans, as observed in sugar beet, tobacco, corn, and other transgenic plants [2,43]. In plants, sucrose synthase (EC 2.4.1.13, SuSy) catalyzes the decomposition of sucrose into fructose and glucose (sugar + UDP ↔ Fructose + UDPG); such a reaction is reversible [44-46]. Different combinations of fructosyltransferases catalyze the synthesis of different fructans [47]. Fructans can be used as storage carbohydrates, they are capable of regulating the sucrose pool size in photosynthetic tissues and during sucrose metabolism, and so on; they release fructose and reduce the water freezing point in tissues via depolymerization of fructans [48]. Therefore, increasing the fructan content is thought to influence the contents of other soluble sugars, total soluble sugars, and sugar metabolic products. For example, the contents of non-structural carbohydrates (glucose, fructose, sucrose, starch, fructan) are significantly higher in trans-*sacB *potatoes than in the wild type [49]. We previously found that the fructan accumulation levels increased in transgenic poplars (D5-20) under drought stress [37]. Transcriptome analysis of transgenic poplar (D5-20) revealed that the differentially expressed genes are enriched in 11 biological pathways linked to glucose metabolism, primarily including starch and sucrose metabolism pathway (KO00500), glucose metabolism pathway (KO00040), and glycolysis/gluconeogenesis pathway (KO00010). The enriched expression of these genes may result in elevated levels of fructan in D5-20. Furthermore, polysaccharide bases in fructan can become inserted between phospholipid bilayer molecules in the cell membrane and can protect lipids and interact with phospholipids to maintain the stability of the cell membrane [50,51].

Plant glycosyltransferase (GT, EC 2.4.x.y) is a key enzyme for glycosylation that catalyzes the synthesis of specific secondary metabolic products and serves as an important component of secondary metabolism. UDP glycosyltransferases (UGTs) catalyzed the transfer of glycosyl groups from UDP saccharides to a variety of receptor molecules, such as carbohydrates (monosaccharides, oligosaccharides, polysaccharides), non-carbohydrates (proteins, lipids, antibiotics, plant hormones, plant toxins, and others), some exogenous substances (herbicides and pesticides), and so on [52]. Glycosyltransferase are therefore thought to be involved in the tolerance of plants to biotic and abiotic stresses. For example, overexpression of *UGT73C5 *in transgenic *Arabidopsis *can improve the resistance against fungal toxins [53]. Moreover, under drought stress, the expression of *UGT74E2 *(UDP-glucosyltransferase) in *Arabidopsis thaliana *can improve the rooting capacity and alter anthotaxy traits by regulating IBA and NAA activities, thereby improving resistance against drought and salt stress [54]. In the present study, numerous glycosyltransferase-related genes were differentially expressed, including six UDP-glucuronosyl/UDP-glucosyl transferase genes (four up-regulated and two down-regulated), four galacturonosyl transferase genes (one up-regulated and down-regulated), one UDP-glucose 4-epimerase/UDP-sulfoquinovose synthase gene (up-regulated), four plant glycosyltransferase genes, two glycosyl hydrolase genes, two glucanase genes, two fructose bisphosphate aldolase genes, and so on (down-regulated). According to KOG functional classification, these genes mainly function in energy production and conversion, carbohydrate transport and metabolism, and cell wall/membrane/envelope biogenesis. The phenomena of up-regulation and down-regulation of similar glycosyltransferase genes may be due to the fact that different protein products are encoded by such genes, or that they have different catalytic substrates. For example, phylogenetic analysis of six UDP-glucuronosyl/UDP glycosyltransferase genes revealed that the amino acid sequences in a single group were identical in two up-regulated genes (Potri.017G052400 and Potri.017G052000), and the other two up-regulated genes were in a group as well. These differences may generate different catalytic activities or different catalytic substrates in the enzyme. Studies of the poplar transcriptome under salt stress have shown some glucose glucosyltransferase genes expressed, suggesting that such genes are involved in the salt stress resistance response in plants [55].

The ERF (ethylene-responsive factor) transcription factors belong to the plant AP2/EREBP (APETALA2/ethylene-responsive element banding protein) superfamily[56] and play important roles in regulating growth, development, and stress response. For example, exogenous *JERF *expression activates the expression of a large number of downstream genes that function in stress resistance and helps increase resistance against salt, drought, and low temperature stress by activating multiple stress-related cis-acting elements in transgenic tobacco plants[57,58]. We previously demonstrated that exogenous *JERF36 *significantly improves the salt tolerance of transgenic poplar (*P. alba × Populus berolinensis*) [59]. Previous physiological greenhouse tests have shown that overexpression of *JERF36 *in D5-20 under salt stress can regulate instantaneous water use efficiency (iWUE) and root growth, thus improving salt resistance [37]. In the present study, 14 AP2/ERF transcription factors in D5-20 were found to be differentially expressed, including 11 that were up-regulated and three that were down-regulated. Among these, seven genes were identified as members of the *ERF *subfamily (six in group B1 and one in group B3) according to genome-wide analysis of the poplar *AP2/ERF *superfamily [39]. In addition, three genes were in the dehydration-responsive element-bonding protein (DREB) subfamily, and one up-regulated gene was in the AP2 subfamily (Group A2), one was in the DREB subfamily (Group A4), and two were down-regulated and in the AP2 subfamily. These findings suggest that the functions of ERF subfamily members are highly conserved, while the functions of genes in the DREB and AP2 subfamilies may be highly divergent. In addition, the differential expression of these AP2/ERF genes may have resulted from the expression of the transferred exogenous gene *JERF36*. Indeed, ERF subfamily genes regulate the activities of downstream genes as well as stress signaling molecules, such as ethylene (ET), jasmonate (JA), and salicylic acid (SA). For example, expression profiling has demonstrated that nine ERF subfamily members are involved in the abscisic acid (ABA), SA, JA, and ET signal transduction pathways, and they are also involved in biotic and abiotic stress responses [60]. Other widely recognized transcription factors involved in plant responses to biotic and abiotic stresses [15], such as WRKY, basic leucine zipper (bZIP) protein, and MYC/MYB, were also identified in our dataset. These factors may participate in ABA and other signal transduction pathways, bind with DNA elements, and induce downstream gene expression in response to stress. Moreover, protein kinases, which act as regulatory factors in plant stress responses, such as receptor-like kinase (RLK), calcium-dependent and calmodulin-independent protein kinase (CDPK), and mitogen-activated protein kinase (MAPK), participate in hormonal signaling transduction processes under various stress conditions. Plant protein kinases can allow signals to be amplified in a step-wise manner through phosphorylation, and they then activate the downstream transcription factors and further induce the expression of resistance genes after they are induced by intracellular second messenger signaling molecules in the signal transduction process. For example, wheat TaMAPK1 can improve TaERF1 activity, promote binding with GCC-box and DRE/CRT elements, and further induce the expression of genes of interest [61,62]. The JA-regulated MAPKK kinase JAM1 and *Arabidopsis thaliana *PKS33 kinase participate in the phosphorylation of the tobacco ERF protein ORC1, and *Arabidopsis *ERF7 further increases ORC1 and ERF7 protein activities [63]. Moreover, the bZIP transcription factor SnRK pathway can be phosphorylated to regulate downstream gene expression [64]. Moreover, recent evidence has shown that protein kinase genes are involved in various biotic and abiotic stress response pathways in an interdependent manner. For example, the CDPK pathway is thought to cross with the MAPK and SnRK pathways [65,66]. Ca^2+^- or Ca^2+ ^receptor-calmodulins (CaMs; such as CaM-like Ca-binding proteins with the EF-hand motif, and Ca^2+^-regulated protein kinases and Ca-binding proteins without the EF-hand motif) play important roles in the signal transduction process in these biotic and abiotic response pathways [67]. In the present study, we detected 20 stress-resistance transcription factors in addition to ERF transcription factors, including five WRKY transcription factors, three zinc-finger transcription factor, three bZip transcription factors, and one MYB transcription factor that were significantly up-regulated. We also detected three MYB transcription factors, one WRKY transcription factor, and two bHLH transcription factor that were significantly down-regulated. We also found that 34 genes encoding serine/threonine protein kinases were up-regulated in the transgenic clones (D5-20), including 17 receptor-like kinases with leucine-rich replicated region, two MAPKs, and two lectin-like kinase; five other serine/threonine protein kinases in D5-20 were down-regulated. A number of genes encoding signaling molecules, including 11 calmodulin binding proteins (nine up- and two down-regulated), 11 ubiquitin ligases (nine up- and two down-regulated), and two up-regulated serine/threonine protein phosphatases, two down-regulated JA amino synthases, and one down-regulated ubiquitin protein were also identified in D5-20 (Additional file 6). These findings suggest that significant, multiple dimensions of the networks involving transcriptional regulation and signal transduction were altered in the D5-20 transcriptome under normal growth condition. Such a phenomenon could largely be attributed to the ectopic expression of transgenes (particularly the transcriptional regulator *JERF36*) followed by the modulation of the activities of numerous genes via transcriptional regulation, phosphorylation, or ubiquitination processes. Nevertheless, the specific mechanisms underpinning this altered gene expression remain unclear and need to be determined.

Vitreoscilla hemoglobin (VHb) is involved in oxygen-related metabolic pathways in the oxygenation state, such as the respiratory chain. This molecule transfers oxygen to the respiratory chain, regulates the activity of terminal oxidase, alters the efficiency of oxidative phosphorylation and metabolic pathways under hypoxic conditions, and promotes cell growth and the expression of some genes [68]. The expression of the *vgb *gene can increase the effective oxygen concentration in a plant and enhance chlorophyll biosynthesis [69,70] and can also promote plant growth [71,72], improve waterlogging resistance [73], and increase the detoxification capacity of plants [74]. We detected 21 differentially expressed genes encoding oxygen metabolic pathway enzymes, including nine (five glutaredoxin-related proteins, two thioredoxins, and two multicopper oxidases) that were up-regulated and 12 (three carbonic anhydrases, two peroxidases, two ferric reduction oxidases, three oxidases, and two iron/ascorbate family oxidoreductases) that were down-regulated. We previously reported that the *Fv*/*Fm *ratio and chlorophyll content in D5-20 were higher than those of D5-0 under flooding stress, suggesting that the expression of *vgb *can help maintain the photosynthetic system stability of a plant through the collection and transport of oxygen, thereby further improving flooding resistance. The large number of differentially expressed oxygen metabolic enzyme genes revealed in the present study may therefore be related to the expression of *vgb *in transgenic poplar.

RNA-Seq of D5-20 also revealed some genes with multiple functions, such as those encoding molecular chaperones, cytochrome P450 transporters, AAA +-ATPase, and chitinase. These genes play essential roles in plant growth and development. For example, DnaJ-like protein is involved in many metabolic processes, functioning as both a molecular chaperone and a regulatory protein [75]. Cytochrome P450 is a large plant protein family that is involved in an array of catalytic reactions, including the synthesis and metabolism of macromolecules (lignin, keratin, and suberin), hormones, signaling molecules, natural pigments, and defensive substances [76]. Transporter proteins are transmembrane proteins that are involved in the uptake, transport, and isolation of ions and small molecules. For example, the uptake and transport of phosphorus in plants relies on phosphorus transporters in the cell membranes of roots [77]. The ATP-binding Cassette (ABC) family proteins play important roles in transmembrane transport of hormones, lipids, carbohydrates, inorganic acids, heavy metals, secondary metabolites, and xenobiotics [78] and are related to chlorophyll synthesis, Fe/S complex formation, and stomatal movement [79]. An AAA+-ATPase gene (*At1g64110*) in *Arabidopsis thaliana *is involved in the abiotic stress response[80]. Chitinase is one of main pathogenesis-related proteins in plants, and it can inhibit the growth of fungi and is involved in the interaction between the host plant and fungal pathogens. Resistance to insects also occurs in plants expressing chitinase genes [81].

*BtCry3A *encodes an endotoxin from *Bt *in Coleoptera and is a type of insecticidal crystal protein (ICP); ICPs are major, active insecticidal ingredients that cannot be directly synthesized in plant cells. Transcriptome analysis has revealed two major biological processes (stress/defense responses and amino acids metabolism) that are influenced by differentially expressed gene in rice expressing a synthetic *Cry1b *gene [82]. However, such phenomena were not observed in the current study of poplar, which may be particularly associated with the potentially complex interactions among genes influenced by multiple transgenes in transgenic D5-20. Additionally, *OC-I *encodes a rice cysteine proteinase inhibitor, and its expression can be induced by JA, ABA, and wounding [83]. The OC-I polypeptide may activate the lipid-based signal transduction pathway, by which plant cells release and convert linolenic acid into the oxylipin signaling molecule JA [84]. Wounding can induce intracellular cascades and plasma membrane depolarization, which opens ion channels, increases intracellular Ca^2+ ^concentrations, and activates MAP kinase and phospholipase A. These processes promote the release of linolenic acid in the cell membrane, transfer linolenic acid into the oxylipin signaling molecule JA, and activate numerous defensive genes [84]. In the present study, we detected a number of esterase genes (up-regulated), JA amino synthase genes (down-regulated), and oligopeptide transporter genes (down-regulated), all of which are possibly involved in the abovementioned processes. We previously demonstrated that the expression of *BtCry3A *and *OC-I *leads to improved insect resistance (mainly to *Plagiodera versicolora*) under both laboratory and field conditions [37,85,86]. However, the effects of the insertion/expression of *BtCry3A *and *OC-I *on the transcriptome of D5-20 remain to be investigated.

Plants face a variety of adverse environmental stresses in the natural growth environment, including biotic stresses (such as fungi, bacteria, viruses, pests, and others) and abiotic stresses (such as high salt, drought, waterlogging, heat, cold, injury, and others). Plants have evolved a systematic defensive mechanism that includes multiple independent and crossed signaling pathways to overcome these adverse environmental conditions. These crossed signaling pathways in plants function during biotic [87], abiotic [88], and combined stress resistance responses [89]. Therefore, the majority of plant traits, such as drought tolerance, salt tolerance, and insect resistance are controlled by multiple genes. These genes interact via signaling pathways in response to biotic and abiotic stresses. Such a phenomenon was also demonstrated in the present study; for example, the transferred *SacB *gene increased the content of fructan, an osmolyte, in transgenic clones, and further altered the expression of glucose metabolism genes. Glycoside metabolism is associated with disease resistance in plants. For example, UDP-glycosyltransferase is involved in resistance against biotic and abiotic stresses through the activities of glycosylated hormones and secondary metabolites in plants [90,91]. Tomato JERFs play an important role in regulatory networks that integrate ET and ABA signaling pathways; JERFs improve salt, drought, and cold tolerance and disease resistance in transgenic plants by regulating the expression of stress-related genes [92]. ERFs play important regulatory roles in molecular responses to hormones (ET), pathogens, cold, drought, high salt, and other stressors [62]. In the present study, the transferred *JERF36 *gene caused the differential expression of a series of transcription factors, regulated gene expression, and led to hormonal changes. There is crosstalk between the ABA-, SA-, JA-, and ET-dependent signaling pathways. These signaling molecules achieve precise regulation of defensive responses in plants through synergistic or antagonistic effects [93]. The transferred *vgb *gene can cause changes in oxygen metabolism in transgenic clones, induce the differential expression of antioxidant protective genes, increase the potential of oxidation stress resistance in transgenic clones, and further improve drought, salt, and reactive oxygen species (ROS) resistance in transgenic clones. A remarkable feature during the early interaction between plants and bacteria is the generation of ROS. ROS can directly help the plant resist pathogenic microorganisms and induce the activation of defensive genes. Therefore, changes in oxygen metabolism can also alter defense responses in plants.

## Conclusions

The stress response in plants is a complicated process associated with multiple genes, multiple signal transduction pathways, and multiple gene expression products. Such processes involve the perception and transduction of stress signals, stress signal identification and transduction in appropriate receptors, and the expression of stress resistance genes. Therefore, comprehensively modify the traits of forest trees requires the transfer of genes in dissimilar metabolic pathways and the synergy of multiple genes. In the present study, transcriptome sequencing of transgenic poplar at the genome-wide level revealed that the transferred exogenous genes caused differential expression of stress (biotic and abiotic) response genes; these genes are distributed in different and associated metabolic pathways. The results of this study provide a theoretical basis for investigating the effects of exogenous genes in transgenic poplar lines. We also detected the differential expression of genes for some unexpected traits, such as genes involved in improved wood properties, nutrient utilization efficiency, and others. However, these results require further observation and verification.

## Methods

### Plant material

The transcriptomes of transgenic (D5-20) and nontransgenic clones (D5-0) of *P. × euramericana *'Guariento' [37] were compared in the current study. The transfected exogenous genes included the following: (1) the *vgb *gene, encoding VHb and related to the improvement of plant adaptive ability to lean oxygen environments; (2) the *SacB *gene, encoding levansucrase and related to plant cell osmotic regulation and the improvement of plant drought resistance; (3) the *BtCry3A *gene, encoding *Bt *insecticidal crystal protein, which confers highly specific resistance to Coleoptera (including poplar longicorn beetles); (4) the *OC-I *gene, encoding rice cysteine protease inhibitors that inhibit the growth of most Coleoptera pests; and (5) the *JERF36 *gene, encoding AP2/EREBP plant transcription factors, which are related to plant stress resistance [37]. The *BtCry3A *and *OC-I *genes formed bivalent genes in the same vector. The neomycin phosphotransferase II gene (*NPT II*) derived from *E. coli *transposon Tn5 was used as a marker, which provided the plants with kanamycin resistance.

### Preparation of cDNA library for RNA-Seq

Cuttings of transgenic (D5-20) and nontransgenic clones (D5-0) grown under the same conditions were selected and cultured in the greenhouse. When the plants grew to approximately 20 cm, with 7-9 leaves, the fourth fully expanded leaves were collected, rapidly frozen in liquid nitrogen, and used for extraction of RNA. Total RNA was extracted using Trizol Reagent (Invitrogen) according to the manufacturer's instructions, and contaminating DNA was digested using DNase I (Promega). The integrity, purity, and quality of total RNA were determined using 1.2% agarose gel electrophoresis and an RNA 6000 Kit (Agilent), and the mRNA was purified using a Dynabeads mRNA Purification Kit (Invitrogen) according to the manufacturer's instructions. The mRNA was randomly broken into fragments after the addition of Fragmentation Buffer (LC-Bio); the mRNA was incubated in 200 μL thin-walled PCR tubes at 94°C for 5 min. The RNA fragments were reverse transcribed into first-strand cDNA, then second-strand cDNA, using random primers and transcriptase. The double-stranded DNA fragments were purified using a QIAquick PCR Purification Kit (Qiagen, Hilden, Germany). The resultant double-stranded cDNA fragments were end-repaired, and an 'A' base was added to their at 3' ends; specific sequencing adaptors were connected to both ends of the DNA fragments. Each DNA fragment was sequenced at a specific joint; 300 bp DNA fragments were recovered through electrophoresis for PCR amplification to enrich the sequencing specimens. The PCR products were purified using a QIAquick Gel Purification Kit (Qiagen). The above procedures were performed according to the instructions for the mRNA-Seq 8-Sample Prep Kit (Illumina).

### Sequencing of transcriptome and processing of sequence data

Libraries of both clones (D5-0 and D5-20) were sequenced using a HiSeq 2000 platform (Illumina) under double-end 100 bp sequencing mode. The quality-reliable sequencing fragments were selected, the poor-quality bases were dynamically removed from the 3' end, and the base fragments with high sequencing quality were ultimately reserved after the primer and adaptor sequences were removed from the raw data and the 3' end quality of sequencing fragments was tested. The remaining sequence fragments were used for subsequent analysis in the RNA-Seq module.

### Mapping reads to the reference genome and annotation

The pretreated sequences were aligned with the poplar reference genomes (http://genome.jgi-psf.org/Poptr1/Poptr1.home.html/). RNA structural features need to be considered with multiple alignments statistical modes during sequence alignment. The alignment parameters and conditions were as follows: (1) Allowable to 2-base unalignment mode; (2) Allowable to maximally optimal 20-base alignment record for each read sequence; (3) Considerable to alternative spicing condition, set the segment length equal to 1/2 read length; (4) Allowable to at most 1 bp unaligned bases in a segment; (5) Set 3 bp insertion and deletion length to the maximum; (6) Allowable to 0-base unalignment in alternative spicing location, namely, complete alignment; (7) Set the min. isoform fraction to 0.15. The above setting indicates that exon A is presumably D higher than that of exon B in sequencing depth if one junction across two exons is overlapped by S sequencing fragments. Exon junctions are not believed to occur currently if the S/D ratio is less than the set minimum isoform fraction (here, 0.15); (8) Set the minimum intron length to 50 bp; (9) Set the maximum intron length to 50,000 bp; and (10) Allowable to 40-base alignment record and 2 bp unaligned bases under junction probing conditions.

### Normalization of gene expression levels

RNA expression levels were calculated using an FPKM indicator. FPKM signifies the fragments per kilo base of transcript per million fragments mapped. The expression levels were calculated using multiple standard methods. In our study, the expression levels and differential gene expression were calculated using standard read mode within reference gene regions using Cufflink software [38].

### Analysis of differentially expressed genes

The expression profiles between repeated experiments (Group A, including four groups, such as Aa + A2) were analyzed in comparison to their differences with three control groups. In difference analysis, the calculation was performed for gene and gene isoform levels. To test the significant variance in expression levels, the log ratios were compared between experimental specimens (conditions) with the log value of a conditional expression level. Fold-changed values between samples were calculated according to FPKM value. The levels of "| log2 Ratio | ≥ 1 and false discovery rate (FDR) ≤ 0.05" were used as the threshold to assess the significance of differential gene expression.

GO functional enrichment analysis was carried out by annotating differentially expressed genes using the Gene Ontology (GO) database and the poplar genome annotation database (http://genome.jgi-psf.org/programs/plants/index.jsf). KOG classifications of differentially expressed genes were performed according to polar genomics annotated information (http://www.phytozome.net/poplar). KEGG pathway analyses of differentially expressed genes were performed using the Kyoto Encyclopedia of Genes and Genomes (KEGG) (http://www.genome.ad.jp/kegg/) database. The enrichment of differentially expressed genes was calculated using Fisher's test and expressed as individual *p *values.

### Annotation and analysis of homologous genes among differentially expressed genes

Homologous protein alignment and annotation analysis were performed for differentially expressed genes using SwissProt and the Uniport non-redundant protein database. The translated protein sequences appropriate to differentially expressed genes were aligned using a Blastp approach and protein databank sequences; the optimally aligned protein sequences with *e *values < 1E-5 were used to derive candidate names of differentially expressed genes/proteins.

### Phylogenetic analysis

Phylogenetic analysis was performed with the AP2/ERF genes with reference to the evolutionary relationships between poplar AP2/ERF genes; the amino acid sequences of UDP-glucuronosyl/UDP glycosyltransferase were completely aligned using default parameters in MEGA5.1 software. The phylogenetic tree was constructed using the neighbor-joining method.

### Quantitative real-time (qRT)-PCR

Cuttings of the transgenic lines D5-20 and the control line D5-0 were cultured in a greenhouse. When the plants grew to approximately 20 cm, with seven to nine leaves, they were exposed to 200 mM NaCl, 20% PEG-6000, or water (water level at 1 cm above the soil surface) for 48 h, and untreated, well-watered plants were used as controls. After each treatment, a mixture of leaves of five plants was collected, frozen immediately in liquid nitrogen, and stored until use. Total RNA was extracted from leaves using an AmbionH Plant RNA Isolation Aid (Applied Biosystems, CA, USA) according to the manufacturer's instructions. Then, cDNA was synthesized using a PrimerScript RT Reagent Kit (TaKaRa, Dalian, China). The qRT-PCR was performed in a LightCycler^® ^480 II Real-time PCR Instrument (Roche, Swiss) with SYBR Green Realtime PCR Master Mix (TaKaRa). Gene-specific primers were designed to amplify 120-130 bp fragments of foreign genes, and parallel PCR was carried out using a gene-specific primer pair for poplar *ACTIN1 *(GenBank Accession XM_002298674), which was used as a reference gene. Primer sequences for the real-time PCR assay of the five genes and *ACTIN1 *are listed in Additional file 7. Five trees were tested per line, and four PCR replicates were performed for each RNA sample. The expression levels of mRNAs were normalized to *ACTIN1 *and were calculated using the 2^-ΔΔCt ^method [94].

## Competing interests

The authors declare that they have no competing interests.

## Authors' contributions

Conceived and designed the experiments: XS BZ QH. Performed the experiments: WZ YC CD. Analyzed the data: WZ YC CD. Contributed reagents/materials/analysis tools: ZH RH YT. Wrote the paper: WZ YC.

## Supplementary Material

Additional file 1 Figure S1: Gene expression density distribution within specimens Click here for file

Additional file 2 **Table S1: List of GO Functional categories for differentially expressed genes**. Click here for file

Additional file 3 **Table S2: List of KOG Functional categories for differentially expressed genes**. Click here for file

Additional file 4 **Table S3: Pathway enrichment analysis for differentially expressed genes**. Click here for file

Additional file 5 Table S4: Main biological pathway of differentially expressed genes Click here for file

Additional file 6 **Table S5: Stress response-related genes**. Click here for file

Additional file 7 **Table S6: Description of primers used in qRT-PCR**. Click here for file

## Declarations

The publication charges this article were funded by the National High-Tech Research and Development Program of China, 863 Program (Grant No. 2011AA100201) and Twelfth Five National Key Technology R&D program (2012BAD01B03)

This article has been published as part of *BMC Genetics *Volume 15 Supplement 1, 2014: Selected articles from the International Symposium on Quantitative Genetics and Genomics of Woody Plants. The full contents of the supplement are available online at http://www.biomedcentral.com/bmcgenet/supplements/15/S1.
