# Supplementary material for: Transcriptome sequencing of transgenic poplar (Populus × euramericana 'Guariento') expressing multiple resistance genes
Source: BMC Genet. 2014 Jun 20;15(Suppl 1):S7. doi: 10.1186/1471-2156-15-S1-S7 (PMC4118631; doi:10.1186/1471-2156-15-S1-S7)
Supplement: Additional file 1 — Figure S1: Gene expression density distribution within specimens [file 1471-2156-15-S1-S7-S1.docx]

**Additional file 1**

**Figure S1: Gene expression density distribution within specimens**


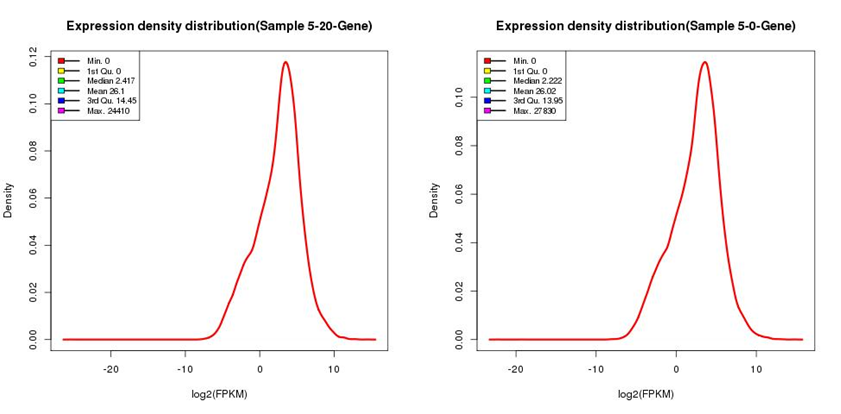
Figure S1: Gene expression density distribution within specimens

We calculated the logarithmic (log2) density distribution of expression levels and the distribution parameters of expression levels (median, mean and percentile, and so on). The expression levels were obtained using the standard FPKM calculation method.
