# Supplementary material for: Transcriptome sequencing of transgenic poplar (Populus × euramericana 'Guariento') expressing multiple resistance genes
Source: BMC Genet. 2014 Jun 20;15(Suppl 1):S7. doi: 10.1186/1471-2156-15-S1-S7 (PMC4118631; doi:10.1186/1471-2156-15-S1-S7)
Supplement: Additional file 5 — Table S4: Main biological pathway of differentially expressed genes [file 1471-2156-15-S1-S7-S5.docx]

Additional file 7

**Table S6 Description of primers used in qRT-PCR.**

| Gene | Forward primer(5’→ 3’) | Reverse primer(5’→ 3’) |
| --- | --- | --- |
| Potri.005G223100 | CTGGCGGACAAGAAGTTATAG | CCACATTCATCAATGGGC |
| Potri.007G138100 | ATCTTGATCTTAACCTGCCTC | GATTACGATGACCCATTAAACG |
| Potri.003G139300 | TGCAAGAATGCCATCTTATGA | GGCATACTTGGAAAGCAG |
| Potri.017G052000 | CTGAAGCGACAACTGGTATT | GCTAAAGGTAGACAACCAGAT |
| Potri.006G055600 | ATGAGAAAGAATGCCAAGAAAT | GCTACTCCTGCAATCTCG |
| Potri.016G128300 | AGCTTCTGCGAGCATATC | CAGCTAAGGTTGTCCATGT |
| Potri.006G105300 | CCCAACCCAAATCATGAC | CGGGATTTCAATTCCATTTCTA |
| Potri.001G202100 | AGCCAGAATATGCTCACG | GAATGGAACCACTTAGACGAT |
| Potri.009G129900 | TCACCTCCTCTGCCTAAC | AGATGTGGGAATTTGGGTAT |
| Potri.001G325800 | CATATTAACGGTTCTCTTGTCC | TGCTACTATACGTGAAGTCATT |
| Potri.006G112500 | CAAGATGGTCGTGTTGATTTC | AGAGAGGATTGAGATCGTGA |
| Potri.T056000 | CTTTAGACTAGACGGAGCTTT | CCAACATTCTTTACTATCCCAT |
| Actin^a^ | TATCTCCTCTGTCTCCGACT | GCATCATCACCTGCAAAC |

a reference gene
