# Supplementary material for: Transcriptome sequencing of transgenic poplar (Populus × euramericana 'Guariento') expressing multiple resistance genes
Source: BMC Genet. 2014 Jun 20;15(Suppl 1):S7. doi: 10.1186/1471-2156-15-S1-S7 (PMC4118631; doi:10.1186/1471-2156-15-S1-S7)
Supplement: Additional file 6 — Table S5: Stress response-related genes. [file 1471-2156-15-S1-S7-S6.docx]

**Additional file 6-Table S5: Stress response-related genes**

| Gene ID | Name | log2.fold_change |  |
| --- | --- | --- | --- |
| Glucose metabolism-related genes | | | |
| Potri.017G052100 | UDP-glucuronosyl and UDP-glucosyl transferase | 1.27807 | △○ |
| Potri.017G052000 | UDP-glucuronosyl and UDP-glucosyl transferase | 1.2506 | △○ |
| Potri.018G140400 | UDP-glucuronosyl and UDP-glucosyl transferase | 1.38641 | △○ |
| Potri.011G060300 | UDP-glucuronosyl and UDP-glucosyl transferase | 1.08074 | △○ |
| Potri.006G178500 | UDP-glucose 4-epimerase/UDP-sulfoquinovose synthase | 1.01757 | △○ |
| Potri.009G044600 | UDP-glucuronosyl and UDP-glucosyl transferase | -1.14232 | △○ |
| Potri.006G055600 | UDP-glucuronosyl and UDP-glucosyl transferase | -1.56611 | △○ |
| Potri.006G030300 | Predicted polypeptide N-acetylgalactosaminyltransferase | 1.03384 | ○ |
| Potri.010G038300 | Probable galacturonosyltransferase-like 9 | -1.04334 | △ |
| Potri.017G036700 | Probable galactinol--sucrose galactosyltransferase 5 | -1.35997 | △ |
| Potri.002G132900 | Probable galacturonosyltransferase-like 1 | -1.89159 | △ |
| Potri.013G095500 | Glycosyltransferase | -1.29721 | △○ |
| Potri.014G029900 | Glycosyl transferase, family 8 - glycogenin | -1.57767 | △○ |
| Potri.001G317600 | Glycosyltransferase | -1.0709 | △○ |
| Potri.010G042000 | Glycosyl transferase, family 8 - glycogenin | -1.31095 | ○ |
| Potri.009G087800 | Glycosyl hydrolase family 10 protein | -1.14891 | △ |
| Potri.002G236200 | Probable xyloglucan endotransglucosylase/hydrolase protein 15 | -1.14909 | △ |
| Potri.001G226100 | Beta-glucosidase, lactase phlorizinhydrolase, and related proteins | -1.40533 | △○ |
| Potri.009G163700 | Glucan endo-1,3-beta-glucosidase 14 | -1.15832 | △ |
| Potri.005G003500 | Beta-1,3 glucanase | -1.26703 | △ |
| Potri.007G015500 | Fructose-biphosphate aldolase | -1.05405 | △○ |
| Potri.004G162400 | Fructose-biphosphate aldolase | -1.02654 | △○ |
| transcription factors | | | |
| Potri.011G115600 | Ethylene-responsive transcription factor 4 | 1.08836 | △ |
| Potri.004G047600 | Ethylene-responsive transcription factor 4 | 1.16823 | △ |
| Potri.004G047500 | Ethylene-responsive transcription factor | 1.78789 | △ |
| Potri.002G043300 | Ethylene-responsive transcription factor 3 | 1.79505 | △ |
| Potri.007G138100 | Ethylene-responsive transcription factor 11 | 1.62556 | △ |
| Potri.006G138800 | Ethylene-responsive transcription factor ERF021 | 1.93424 | △ |
| Potri.001G154200 | Ethylene-responsive transcription factor 2 | 1.84779 | △ |
| Potri.005G223100 | Ethylene-responsive transcription factor 13 | 2.06153 | △ |
| Potri.005G148400 | AP2-like ethylene-responsive transcription factor ANT | 2.12717 | △ |
| Potri.018G047300 | Ethylene-responsive transcription factor ERF016 | 2.98996 | △ |
| Potri.006G218200 | Ethylene-responsive transcription factor ERF016 | 2.7483 | △ |
| Potri.006G105300 | Probable WRKY transcription factor 13 | 1.58047 | △ |
| Potri.006G105300 | WRKY transcription factor 55 | 2.16986 | △ |
| Potri.016G128300 | WRKY2_ARATH Probable WRKY transcription factor 2 | 1.28595 | △○ |
| Potri.014G096200 | WRKY transcription factor 55 | 1.42392 | △ |
| Potri.003G138600 | Probable WRKY transcription factor 52 | 1.93539 | △ |
| Potri.014G017300 | FOG: Zn-finger | 1.31234 | △○ |
| Potri.010G187900 | CCCH-type Zn-finger protein | 1.01461 | △○ |
| Potri.009G143700 | FOG: Zn-finger | 2.94912 | △○ |
| Potri.006G241700 | Transcription factor, Myb superfamily | 1.43956 | △○ |
| Potri.014G099900 | Ethylene-responsive transcription factor ERF042 | -1.1252 | △ |
| Potri.003G139300 | Ethylene-responsive transcription factor ERF053 | -1.30898 | △ |
| Potri.001G155700 | Ethylene-responsive transcription factor ERF038 | -1.77834 | △ |
| Potri.014G111200 | Transcription factor, Myb superfamily | -1.15798 | △○ |
| Potri.019G081500 | Transcription factor, Myb superfamily | -1.32369 | △○ |
| Potri.008G081800 | GATA-4/5/6 transcription factors | -1.00721 | △○ |
| Potri.014G111900 | Transcriptional corepressor Atrophin-1/DRPLA | -2.95852 | △○ |
| Potri.007G023600 | Transcription factor bHLH63 | -1.10778 | △ |
| Potri.008G161800 | Transcription factor bHLH92 | -1.13714 | △ |
| protein kinase | | | |
| Potri.002G087600 | Dual specificity; serine/threonine and tyrosine kinase | 1.02146 | ○ |
| Potri.005G173300 | Dual specificity; serine/threonine and tyrosine kinase | 1.51395 | ○ |
| Potri.006G080500 | Serine/threonine protein kinase | 1.06814 | △○ |
| Potri.010G109600 | Serine/threonine protein kinase | 1.66183 | ○ |
| Potri.018G057700 | Serine/threonine protein kinase | 1.16205 | △○ |
| Potri.019G078300 | Serine/threonine protein kinase | 1.27645 | △○ |
| Potri.006G235000 | Serine/threonine protein kinase | 1.25063 | △○ |
| Potri.002G049400 | Serine/threonine protein kinase | 1.40628 | △○ |
| Potri.008G195800 | Serine-threonine protein kinase | 1.30473 | ○ |
| Potri.019G052500 | Serine-threonine protein kinase | 1.31382 | ○ |
| Potri.010G031800 | Serine-threonine protein kinase | 1.44066 | ○ |
| Potri.T058000 | Serine-threonine protein kinase | 1.51051 | ○ |
| Potri.011G028500 | Serine/threonine protein kinase | 1.08237 | △○ |
| Potri.006G104300 | FOG: Leucine rich repeat | 1.30847 | △○ |
| Potri.016G126300 | FOG: Leucine rich repeat | 1.02984 | △○ |
| Potri.006G220100 | FOG: Leucine rich repeat | 1.07878 | △○ |
| Potri.002G019900 | FOG: Leucine rich repeat | 1.5972 | △○ |
| Potri.002G258500 | FOG: Leucine rich repeat | 1.09749 | ○ |
| Potri.014G008200 | FOG: Leucine rich repeat | 1.37791 | ○ |
| Potri.005G235100 | FOG: Leucine rich repeat | 1.19312 | ○ |
| Potri.014G024400 | Serine/threonine protein kinase | 1.24125 | △○ |
| Potri.008G211800 | FOG: Leucine rich repeat | 1.86704 | ○ |
| Potri.008G150200 | FOG: Leucine rich repeat | 1.94011 | △○ |
| Potri.001G202100 | FOG: Leucine rich repeat | 2.49319 | ○ |
| Potri.012G071100 | Serine/threonine protein kinase | 1.86305 | △○ |
| Potri.010G155200 | Serine/threonine protein kinase | 1.58129 | △○ |
| Potri.014G068700 | Serine/threonine protein kinase | 1.2064 | △○ |
| Potri.006G220100 | Serine/threonine protein kinase | 1.08131 | △○ |
| Potri.018G101300 | Serine/threonine protein kinase | 1.42713 | △○ |
| Potri.004G059100 | Serine/threonine protein kinase | 2.44202 | △○ |
| Potri.004G014700 | Serine/threonine protein kinase | 1.66299 | △○ |
| Potri.019G086200 | Serine/threonine protein kinase | 1.7729 | △○ |
| Potri.010G092000 | MEKK and related serine/threonine protein kinases | 1.11975 | △○ |
| Potri.005G139300 | MEKK and related serine/threonine protein kinases | 2.73094 | △○ |
| Potri.017G141400 | Serine/threonine protein kinase | -1.23613 | ○ |
| Potri.T023700 | Serine/threonine protein kinase | -1.22612 | △○ |
| Potri.017G047500 | Serine/threonine protein kinase | -1.01343 | ○ |
| Potri.011G058300 | Serine/threonine protein kinase | -1.18301 | △○ |
| Potri.009G129900 | Serine/threonine protein kinase | -1.84109 | ○ |
| Stress signal transduction related genes | | | |
| Potri.001G411800 | Ca^2+^-binding protein (centrin/caltractin), EF-Hand superfamily protein | 1.3981 | ○ |
| Potri.001G411800 | Ca^2+^-binding protein (centrin/caltractin), EF-Hand superfamily protein | 1.3981 | ○ |
| Potri.T055700 | Calmodulin and related proteins (EF-Hand superfamily) | 1.02622 | △○ |
| Potri.019G026900 | Calmodulin and related proteins (EF-Hand superfamily) | 1.12147 | △○ |
| Potri.019G026900 | Calmodulin and related proteins (EF-Hand superfamily) | 1.26418 | △○ |
| Potri.002G001400 | Calmodulin and related proteins (EF-Hand superfamily) | 1.91099 | △○ |
| Potri.006G112500 | Calmodulin and related proteins (EF-Hand superfamily) | 2.02549 | △○ |
| Potri.T055600 | Calmodulin and related proteins (EF-Hand superfamily) | 1.02334 | △○ |
| Potri.019G027000 | Calmodulin and related proteins (EF-Hand superfamily) | 1.08427 | △ |
| Potri.009G049600 | Ubiquitin-protein ligase | 1.33584 | △○ |
| Potri.001G254500 | Ubiquitin-protein ligase | 1.25271 | △○ |
| Potri.009G034800 | FOG: Predicted E3 ubiquitin ligase | 1.44078 | △○ |
| Potri.005G036800 | FOG: Predicted E3 ubiquitin ligase | 1.40283 | △○ |
| Potri.008G219200 | FOG: Predicted E3 ubiquitin ligase | 1.31222 | △○ |
| Potri.018G098000 | FOG: Predicted E3 ubiquitin ligase | 2.37548 | △○ |
| Potri.017G122800 | E3 ubiquitin-protein ligase | 1.03241 | △○ |
| Potri.017G122800 | E3 ubiquitin-protein ligase | 1.03241 | △○ |
| Potri.003G162400 | E3 ubiquitin-protein ligase | 1.06904 | △○ |
| Potri.001G381000 | Serine/threonine protein phosphatase | 1.22026 | △○ |
| Potri.006G232700 | Serine/threonine protein phosphatase | 2.35692 | △○ |
| Potri.T056000 | Calmodulin and related proteins (EF-Hand superfamily) | -1.16366 | △ |
| Potri.019G029000 | Calmodulin and related proteins (EF-Hand superfamily) | -1.6951 | △ |
| Potri.014G095500 | Jasmonic acid-amido synthetase JAR1 | -1.15105 | △ |
| Potri.002G168200 | Jasmonic acid-amido synthetase JAR1 | -1.07894 | △ |
| Potri.004G175000 | Ubiquitin-protein ligase | -1.09039 | △○ |
| Potri.014G075000 | FOG: Predicted E3 ubiquitin ligase | -1.02488 | △○ |
| Potri.011G026600 | Ubiquitin and ubiquitin-like proteins | -1.03385 | △○ |
| Oxygen metabolism-related genes | | | |
| Potri.006G212700 | Glutaredoxin-related protein | 1.55252 | ○ |
| Potri.008G016100 | Glutaredoxin-related protein | 1.12525 | ○ |
| Potri.001G448300 | Glutaredoxin-related protein | 1.63387 | △○ |
| Potri.004G049800 | Glutaredoxin and related proteins | 2.06071 | △○ |
| Potri.001G325800 | Glutaredoxin and related proteins | 4.4948 | △○ |
| Potri.010G060300 | Thioredoxin, nucleoredoxin and related proteins | 1.0726 | △○ |
| Potri.010G059700 | Thioredoxin, nucleoredoxin and related proteins | 1.17459 | △○ |
| Potri.012G126400 | Multicopper oxidases | 1.23446 | △○ |
| Potri.015G127200 | Multicopper oxidases | 1.6267 | △○ |
| Potri.015G075900 | Predicted carbonic anhydrase involved in protection against oxidative damage | -1.63885 | △○ |
| Potri.015G076000 | Predicted carbonic anhydrase involved in protection against oxidative damage | -1.61882 | △○ |
| Potri.009G129900 | Carbonic anhydrase | -1.74459 | △○ |
| Potri.005G108900 | Peroxidase 64 | -1.11304 | △ |
| Potri.013G156500 | Peroxidase 52 | -1.42624 | △ |
| Potri.015G083200 | Ferric reductase, NADH/NADPH oxidase and related proteins | -1.76266 | △○ |
| Potri.017G142800 | Ferric reductase, NADH/NADPH oxidase and related proteins | -1.22097 | △○ |
| Potri.001G378400 | Iron/ascorbate family oxidoreductases | -1.43959 | △○ |
| Potri.002G040700 | Iron/ascorbate family oxidoreductases | -1.2872 | △○ |
| Potri.002G088600 | Sulfite oxidase, molybdopterin-binding component | -1.16035 | △○ |
| Potri.012G001500 | Alternative oxidase 1c, mitochondrial | -1.033 | △ |
| Potri.001G248700 | Multicopper oxidases | -1.24799 | ○ |
| Ion transport-related genes | | | |
| Potri.010G165800 | Predicted small molecule transporter | 1.02835 | △○ |
| Potri.010G165300 | Predicted small molecule transporter | 1.55841 | △○ |
| Potri.001G470200 | Amino acid transporters | 1.54951 | △○ |
| Potri.009G132100 | Amino acid transporters | 1.38495 | △○ |
| Potri.004G172800 | Amino acid transporters | 1.16679 | △○ |
| Potri.001G095600 | Multidrug resistance-associated protein/mitoxantrone resistance protein, ABC superfamily | 1.15631 | △○ |
| Potri.015G081300 | High affinity nitrate transporter 2.5 | -1.08058 | △ |
| Potri.015G081500 | High affinity nitrate transporter 2.5 | -1.30243 | △ |
| Potri.018G040600 | H+/oligopeptide symporter | -1.53615 | △○ |
| Potri.018G040500 | H+/oligopeptide symporter | -1.51893 | △○ |
| Potri.018G040700 | H+/oligopeptide symporter | -1.74653 | △○ |
| Potri.006G115000 | Pleiotropic drug resistance proteins (PDR1-15), ABC superfamily | -1.01847 | △○ |
| Potri.014G078000 | Predicted transporter/transmembrane protein | -1.61306 | △○ |
| Potri.013G030900 | Amino acid transporters | -2.34755 | △○ |
| Potri.004G083300 | K+-channel ERG and related proteins, contain PAS/PAC sensor domain | -1.05813 | △○ |
| Potri.015G026700 | S-type anion channel | -1.66142 | △ |
| Potri.015G026800 | S-type anion channel | -2.01584 | △ |
| Other functional genes | | | |
| Potri.006G056400 | Molecular chaperone (DnaJ superfamily) | 1.49835 | ○ |
| Potri.016G051800 | Molecular chaperone (DnaJ superfamily) | 1.07173 | ○ |
| Potri.001G043100 | Molecular chaperone (DnaJ superfamily) | 1.06912 | △○ |
| Potri.008G095000 | Multifunctional chaperone (14-3-3 family) | 1.02348 | △○ |
| Potri.017G147000 | Copper chaperone | 1.31246 | △○ |
| Potri.016G006600 | Copper chaperone | 1.04402 | △○ |
| Potri.002G032800 | Copper chaperone | 2.27449 | ○ |
| Potri.009G109700 | Cytochrome P450 | 1.40955 | △○ |
| Potri.007G084800 | Cytochrome P450 CYP2 subfamily | 1.56989 | △○ |
| Potri.013G125300 | Cytochrome P450 CYP2 subfamily | 1.26651 | △○ |
| Potri.004G018800 | Cytochrome P450 CYP2 subfamily | 1.57763 | △○ |
| Potri.001G331200 | AAA+-type ATPase | 1.47481 | ○ |
| Potri.012G086400 | AAA+-type ATPase | 1.51402 | ○ |
| Potri.001G331200 | AAA+-type ATPase | 1.34222 | ○ |
| Potri.015G084900 | AAA+-type ATPase | 1.69128 |  |
| Potri.017G002200 | Chitinase | 1.33003 | ○ |
| Potri.004G182100 | Predicted chitinase | 1.27425 | △○ |
| Potri.001G177400 | esterase/lipase 5 | 1.09491 | △ |
| Potri.019G024400 | esterase/lipase | 1.44575 | △ |
| Potri.018G089300 | esterase/lipase | 1.18424 | △ |
| Potri.017G102100 | esterase/lipase 7 | 1.95394 | △ |
| Potri.006G266500 | Probable pectinesterase 68 | 1.86456 | △ |
| Potri.013G051000 | esterase/lipase LTL1 | 1.59685 | △ |
| Potri.013G115900 | esterase/lipase | 1.82829 | △ |
| Potri.019G008300 | esterase/lipase | 1.79238 | △ |
| Potri.019G005600 | esterase/lipase | 1.844 | △ |
| Potri.001G469600 | Molecular chaperone (DnaJ superfamily) | -1.07797 | ○ |
| Potri.013G055600 | Zuotin and related molecular chaperones (DnaJ superfamily), contains DNA-binding domains | -1.34579 | ○ |
| Potri.014G141500 | Molecular chaperone (small heat-shock protein Hsp26/Hsp42) | -1.07619 | ○ |
| Potri.014G037400 | Cytochrome P450 CYP2 subfamily | -1.48393 | △○ |
| Potri.014G037700 | Cytochrome P450 CYP2 subfamily | -1.22176 | △○ |
| Potri.T115400 | Cytochrome P450 CYP2 subfamily | -1.30966 | △○ |
| Potri.010G139300 | Cytochrome P450 CYP4/CYP19/CYP26 subfamilies | -1.08083 | △○ |
| Potri.T115400 | Cytochrome P450 CYP2 subfamily | -1.12849 | △○ |
| Potri.014G072000 | Cytochrome P450 CYP4/CYP19/CYP26 subfamilies | -1.67464 | ○ |
| Potri.004G235400 | Cytochrome P450 CYP4/CYP19/CYP26 subfamilies | -1.6444 | ○ |

△, the genes were annotation in SwissProt protein database; ○,the genes were annotation in KOG database; △○, the genes were annotation both in SwissProt and KOG database .
